# Modeling-Enabled Systems Nutritional Immunology

**DOI:** 10.3389/fnut.2016.00005

**Published:** 2016-02-16

**Authors:** Meghna Verma, Raquel Hontecillas, Vida Abedi, Andrew Leber, Nuria Tubau-Juni, Casandra Philipson, Adria Carbo, Josep Bassaganya-Riera

**Affiliations:** ^1^Nutritional Immunology and Molecular Medicine Laboratory (www.nimml.org), Biocomplexity Institute, Virginia Tech, Blacksburg, VA, USA; ^2^The Center for Modeling Immunity to Enteric Pathogens, Biocomplexity Institute, Virginia Tech, Blacksburg, VA, USA; ^3^BioTherapeutics Inc, Blacksburg, VA, USA

**Keywords:** nutritional immunology, nutrition, systems biology, informatics, computational modeling, big data, complex systems

## Abstract

This review highlights the fundamental role of nutrition in the maintenance of health, the immune response, and disease prevention. Emerging global mechanistic insights in the field of nutritional immunology cannot be gained through reductionist methods alone or by analyzing a single nutrient at a time. We propose to investigate nutritional immunology as a massively interacting system of interconnected multistage and multiscale networks that encompass hidden mechanisms by which nutrition, microbiome, metabolism, genetic predisposition, and the immune system interact to delineate health and disease. The review sets an unconventional path to apply complex science methodologies to nutritional immunology research, discovery, and development through “use cases” centered around the impact of nutrition on the gut microbiome and immune responses. Our systems nutritional immunology analyses, which include modeling and informatics methodologies in combination with pre-clinical and clinical studies, have the potential to discover emerging systems-wide properties at the interface of the immune system, nutrition, microbiome, and metabolism.

## Introduction

The knowledge that food affects health was first mentioned in the writings of ancient Egyptians and Indians (1–3). Around 2,500 years ago, Hippocrates, stated “Let food be your medicine and medicine be your food” (4). Modern nutritional immunology dates back to the eighteenth century, when the explanation of lymphoid tissue atrophy in malnourished population in England (1, 3) suggested an association between nutritional status and immune function. Epidemiological and clinical data also suggest that nutritional deficiencies of essential dietary components, such as vitamins and micronutrients, alter immune competence and increase the risk of infection. The deficiency of adequate macronutrients and selected micronutrients, such as zinc, selenium, iron, copper, and vitamins A, B-6, C, E, leads to immune deficiency-related infections in children (5, 6). Micronutrient deficiencies affect innate immune responses as well as adaptive cellular immune responses (7). The immune response is dependent on the nutritional components of food intake, which modulates the induction of regulatory versus effector response at the gut mucosal level (3). However, recent studies (3) suggest that the current immune deficiency cases are also the result of increased stress, increased caloric intake, obesity, autoimmunity, allergic disorders, and an aging population, which do not necessarily relate to under-nutrition. Thus, unbalanced nutrition, unhealthy lifestyle choices, limited physical activity, and the effect of the environment, in general, compromise the host immune response, thereby increasing susceptibility to a wide range of diseases. The field of nutritional immunology primarily focuses on the role of diet and its nutritional contents in disease prevention. However, advancement in the field of nutritional immunology has not been investigated through the point of view of a massively interacting system of interconnected networks, which includes four key players – nutrition, microbiome, metabolism, and the immune system. Recent evidence (8) also suggests the involvement of diet and the role of composition of microbiota in reduced risk of Parkinson’s disease (PD). There are findings that support the role of altered gut microbiome involved in influencing the activity of enteric neurons in PD patients (8). Although it is still unclear, the neuroendocrine system can be considered as an important part of the massively interacting multistage networks that define health and wellness. An understanding of the interaction between networks can help design better strategies for primary prevention for diseases, such as PD, which show the involvement of gut–brain axis in the disease pathogenesis (8). The investigation from the above-mentioned point of view requires modeling tools, informatics techniques, and major computational resources in order to gain a better understanding of the mechanisms by which the four key players interact, to delineate health and disease. The vast aspects of this interconnected network operate on the basis of complex regulatory networks that can be analyzed in a well-defined manner using mathematical and computational modeling. The recent modeling frameworks applied include the use of (1) ordinary differential equations (ODEs) that are used for cancer immunology, natural killer cell responses, B cell responses (naïve and memory), T regulatory cell dynamics and T cell responses; (2) partial differential equations are used for modeling age-structured and spatiotemporal models; (3) stochastic differential equations account for noise and sporadic events, (4) agent-based models account for probabilistic uncertainty in biological interaction (9), and (5) advanced machine-learning algorithms that correlate cellular and molecular events to changes in health and disease outcomes. In the following sections, we dissect the essence of interactions between the four key players following with the review of technological advances in the field of nutritional immunology research and development.

## The Interplay Between Diet, Microbiome, Metabolism, and Immune Response

The proper nutritional supply during the period of gestation, neonatal maturation, and weaning contributes toward the development of balanced immune responses. With an increasing shift in our focus toward using dietary interventions to regulate the host defense, it is important to understand the effect of overall nutrition derived from these interventions. The nutritional quality of the wholesome diet modulates the interactions between the immune system, microbiome, and metabolism.

**Figure 1:**
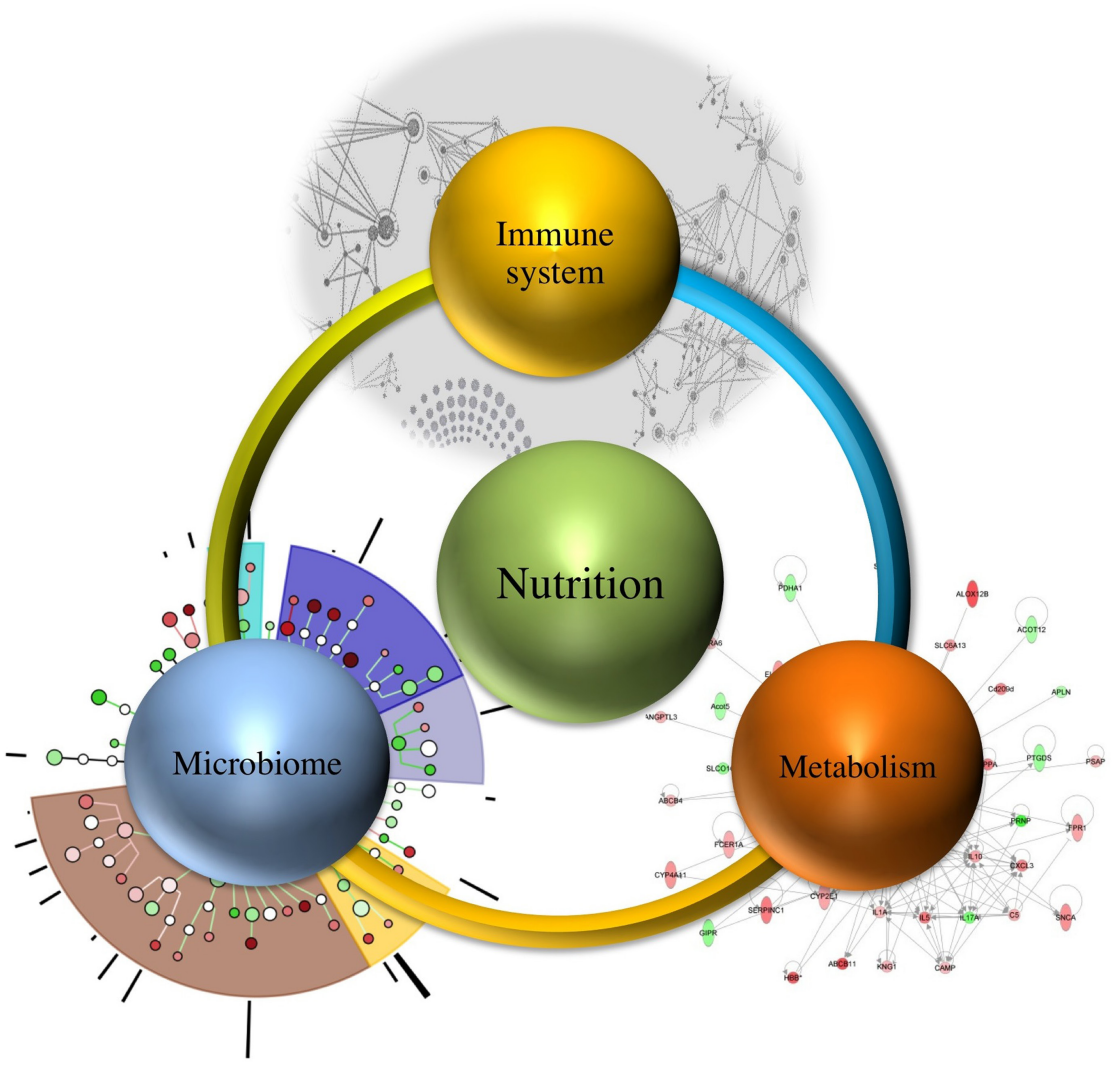
**Systems-wide interactions between nutrition, immune system, microbiome, and metabolism**.

It is estimated that demand of feeding a population will increase up to nine billion people needing food by 2050, which necessitates the need for devising methods that not only meet the demand but also ensure continuous wholesome food supply (10). Therefore, understanding the relationship between immune system, microbiome, and metabolism regulated by nutrition (as shown in Figure 1) will assist in targeting one component at a time, while recognizing their systems-wide effects. This would lead to identification of emerging properties of this complex system and utilization of the newly derived information and knowledge for improved health outcomes.

### Microbiome, and Its Interaction with Nutrition, Immune Response, and Metabolism

Microbes are important components of the human ecosystem, and they account for approximately 100 trillion, including both the ones residing outside as well as inside the human body (11, 12). The gut microbiome is a key player in regulating the defense responses and metabolism, thereby contributing toward shaping the immune responses (regulatory or effector) and aiding in the maturation of the immune system. The various physiological factors responsible for differences in genetic elements of the microbiome within a host include diet, geographical location, and environmental interaction (13). The interactions between the gut microbiome, immune system, metabolism, and nutrition are crucial determinants of health outcomes. However, their systems-wide mechanisms of interaction remain largely unknown. The advent of computational modeling and informatics provides the technology to integrate and comprehensively analyze the multiscale interactions within such networks. Thus, a systems-wide approach can provide significant insights into nutritional regulation of this holistic network, without unnecessarily resorting to reductionism.

#### Interplay between Microbiome and Nutrition

Diet and nutritional status are the key players in defining the composition and function of the gut microbiome as well as the host immune response. The nutritional value of food is influenced by microbial content inside a person’s gut. A study by Turnbaugh et al. (14) demonstrated that transfer of microbiota from mice with diet-induced obesity to lean germ-free mice, showed a greater fat deposition in the lean mice versus the lean ones with transplants from the lean donors. Another study by Turnbaugh et al. (15) explored the use of humanized gnotobiotic mice wherein adult human fecal microbial communities were transplanted into germ-free mice to show the effect of Western diet on the varying bacterial colonization in adult mice. The switch from regular to Western diet showed the colonization of *Firmicutes* Bacilli along the length of the gut, leading to increased adiposity. The effect was shown to be reversible, based on the combinations of recipient–donor diets. These studies (14–17) show that dietary intake influences the composition and activity of the gut microbiome in humans. Specific strains of bacteria have been implicated in the regulation of the intestinal homeostasis, which deliver regulatory signals to the epithelium and the mucosal immune system (18). Even a short-term consumption of animal-based diet versus plant products has a differential effect on the bacterial colonization inside the gut (19). A recent study by Daniel et al. (20) showed that a high-fat diet-induced changes in the chemical composition of cecum thereby causing changes in the bacterial physiology and metabolism. Furthermore, the distance between diet-dependent clusters of the microbial composition was higher than microbiota-driven clusters, thereby illustrating how diet can alter the microbiota profiles to a higher extent than bacterial composition. The effect of diet on the composition of every individual’s microbiome is shown to be individual-specific at the operational taxonomic units and stable over a period of time in a healthy adult (21). However, as the individual ages, there is an extreme variability observed in composition of the core microbiota. Furthermore, there are other environmental factors, such as body weight, physical activity, and exposure to toxins, which also play an important role on the composition of microbiota. A comprehensive understanding of nutritional quality of the dietary interventions (22–24) that modulate the components of the gut microbiota and mucosal immune responses can prove useful for maintenance of health. A systems-level framework that integrates various *in vitro* and *in vivo* models, including human data, can facilitate the systems-wide mechanistic insights (25).

#### Role of the Microbiome in Shaping a Healthy Immune System

**Figure 2:**
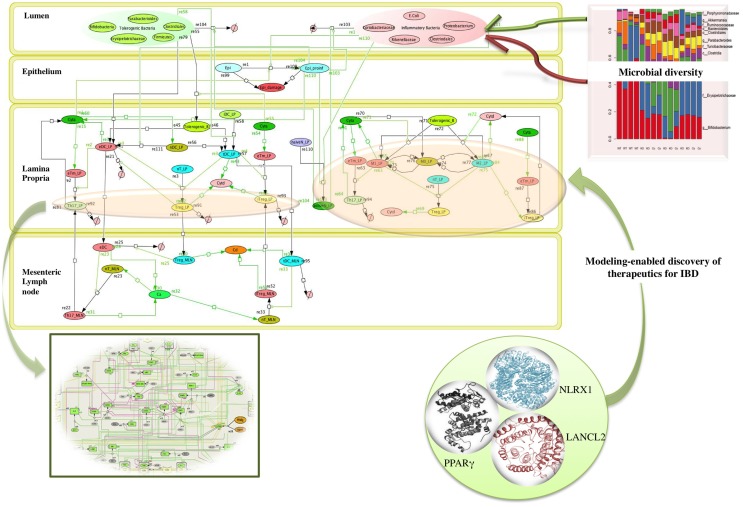
**Network topology of model illustrating mucosal responses to inflammatory bowel disease with novel therapeutic targets in the in-view**. Systems biology markup language (SBML) compliant network of interactions between commensal and foreign bacteria on the cellular immune components is created using CellDesigner.^1^ The bigger panel in the figure represents the different compartments of the gut that includes the lumen, epithelium, lamina propria, and mesenteric lymph node. The red and the green objects represent foreign bacteria and commensal bacteria, respectively, found inside the lumen of the gut. The stacked column bar graph depicts the relative abundances and distribution of the various microbial communities present inside the gut. The imbalance between the red (foreign bacteria) and green (commensal bacteria) objects represents the dysbiosis inside the lumen in inflammatory bowel disease (IBD). The dysbiosis in the lumen causes the activation of inflammatory cytokines (shown by green arrows) in the lamina propria. The three in-view of the molecules represents the modeling-enabled discovery of lanthionine synthetase cyclase-like 2 (LANCL2), nod-like receptor-X1 (NLRX1), and peroxisome proliferator-activated receptor γ (PPAR γ) that are the targets for therapeutic intervention for treatment of IBD. The rectangular in-view represents the complex intracellular signaling pathways and transcriptional factors controlling T cell network (124). ^1^http://www.celldesigner.org/

Microbiome plays a crucial role in shaping the functions of the immune system thereby providing a protective mechanism to fight against infection. The commensal bacteria help in maintaining the balance with the foreign (often pathogenic) bacteria, by modulating the components of host innate immune system. A dysregulation of homeostasis between host and gut microbes leads to dysbiosis, which can give rise to pathogenic states, such as inflammatory bowel disease (IBD) (21, 26) as shown in the network model of IBD in Figure 2. A change in the composition of gut microbes has been associated with development of asthma in animal models. A recent study by Arrieta et al. (27) demonstrated that infants who exhibit transient gut microbial dysbiosis during the early days of life are at high risk of asthma. The inoculation of germ-free mice with the bacterial genera *Lachnospira, Veillonella, Faecalibacterium, and Rothia* (missing in children at high risk asthma), ameliorated the airway inflammation in germ-free adult offspring (27). The study elucidates the role of gut microbiome in protecting the body against asthma. A recent study by Fonseca et al. (28) showed that during the post resolution of infection stage from *Yersinia pseudotuberculosis*, the signals derived from the gut microbiota aided in the maintenance of inflammatory mesentery remodeling and restoration of mucosal immunity. However, persistent disruption of communication between tissues and the immune system, following the clearance of an acute infection represents a point beyond which tissue immunity is compromised for the long term. The intestinal immune system, thus, plays an important role in maintaining the balance of commensal and foreign microorganisms inside the gut along with keeping the diversity of the commensal microorganisms. However, due to high bacterial densities inside the gut, the task is challenging as compared to other organs and tissues. The immune system has adopted certain ways, such as immunological tolerance, by diverting various resources to segregate the microbiome on the luminal side of the epithelial barrier (29). The production of mucus by the goblet cells residing in the intestinal epithelium creates a protective layer that separates the commensal and pathogenic bacteria. This protective zone aids in the maintenance of the symbiotic relationship with the lumen microbiota. The compartmentalization of pathogenic bacteria includes the trapping of bacteria inside the mucus layer, complement-associated bacterial killing, and promotion of phagocytosis of bacteria that invade the epithelial barrier (30). Another mechanism that promotes the segregation of the bacterial colonies is the secretion of antimicrobial proteins. Activation of the intestinal epithelial cells triggers the expression of antimicrobial proteins that provides a protective mechanism against the invasion of the pathogenic bacteria into the host tissues (30). The production of IgA also helps in the maintenance of the symbiotic relationship, but the mechanisms of protection by IgA remain unclear.

Overall the changes in the composition of the gut microbiome can modulate the induction of regulatory versus effector immune responses. Probiotics have been shown to beneficially modulate the intestinal ecosystem. Another group of non-digestible food ingredients is the prebiotics that favors the growth of health-promoting bacteria, proving beneficial to the host (18, 22, 31). A large group of prebiotics comprises the carbohydrates that are indigestible by human digestive enzymes, such as resistant starches. The bacterial community inside the intestinal mucosal surface ferments the undigested fibers to generate lipid molecules, such as oleic acid and conjugated linoleic acids (CLAs), and short chain fatty acids (SCFAs), such as acetate, propionate, and butyrate, that influences the colonic mucosal growth and intestinal permeability that enhances the gastrointestinal health (31, 32). A study by Bassaganya-Riera et al. (32) compared the anti-inflammatory efficacy and studied how different dietary soluble fibers and resistant starch influence regulatory T cells (Tregs), colonic peroxisome proliferator-activated receptor γ (PPAR γ), and interferon gamma (IFN-γ) to suppress gut inflammation. Thus, both probiotics and prebiotics can influence the composition of the intestinal microflora and alter the metabolic composition of the microbiome (22, 32, 33) In fact, in cases of dysbiosis, the possibility of manipulating the gut bacterial composition by using probiotic bacteria has already been explored as a promising therapeutic intervention against IBD (22). The study (22) investigated the molecular mechanism underlying the anti-inflammatory effect of probiotic bacteria using a mouse model of colitis. The results from the study (22) showed that probiotic bacteria modulated microbial diversity of the gut and favored the production of CLA that targeted myeloid cells PPAR γ to suppress colitis. The network topology model of IBD shown in Figure 2 refers to IBD condition caused due to dysbiosis and highlights the complexity of the multi-network, multiscale mucosal immune responses that influences initiation, progression, and outcome of the disease.

### Metabolism and Its Effect on Immune System and Microbiome

Multiple bacterial genomes modulate the metabolic reactions inside the body exemplified by the production of SCFAs, an essential component of host health. Humans lack enzymes required for digestion of dietary fibers (34). The microbial community inside the gut ferments these undigested carbohydrates for energy storage. As mentioned in the previous section, the fermentation results in a wide variety of lipid molecules, including oleic acid and SCFAs (34) such as butyrate, propionate, acetate, that provide the colon with energy required during metabolic demands as well as regulatory signals that help in the maintenance of homeostasis. Along with being a local nutrient source for colonocytes, SCFAs regulate energy homeostasis by stimulating lectin production in adipocytes as well as glucagon-like peptide secretion by the intestinal endocrine cells. The SCFAs also regulate neutrophil function and migration, inhibit inflammatory cytokine-induced expression of vascular cell adhesion molecule-1, and increase the expression of tight junction proteins in the colon epithelia. Overall, they affect a wide range of host processes, including energy utilization, host–microbe signaling, epithelial cell integrity, and gut mobility (35). Oleic acid is a commonly found dietary component and is also a microbial metabolism product. Increased concentrations of oleic acid are found within *Parabacteroides* (36), and oral treatment with commensal *Parabacteroides distasonis* has been shown to significantly reduce the severity of intestinal inflammation in murine models of acute and chronic colitis (37). Thus, it is important to understand whether the diet-derived products of microbial metabolism are released under similar conditions in presence of varying food substrates that may include proteins, carbohydrates, and fat. The host metabolome is a rich resource for studying metabolic function of the gut microbiome. Multi-omic data integration through modeling can facilitate a comprehensive mechanistic understanding of how dietary and microbial components in the gut modulate immune responses. These technologies are at the very core of advancing nutritional-based precision medicine interventions and moving from understanding single nutrients to understanding the impact of nutrition at the systems level.

### Nutrition – A Key Player in the Immune System–Diet Interaction Network

The nutritional status of an individual is a key determinant of the susceptibility of the immune system to infection and disease (10, 38). During infection, the host requirements for energy substrates and nutrients rapidly increase in the presence of invading microorganisms or in any immune-mediated disease that involves proliferation of immune cell subsets. However, it is widely known that infectious agents reduce the motivation for voluntary food intake due to the stimulation of leukocytes to produce inflammatory cytokines. The immune cells use these cytokines to convey information to other physiological systems, including the brain that modulates the food intake (39, 40). The increased metabolic demands are utilized to raise the body temperature (for e.g., in fever) (41) required for the proliferation of the immune cells in the course of elimination of an infectious pathogen. The growth, survival, and differentiation of the activated immune cells depend on glucose metabolism as a source of energy, which has a huge impact on our health (42). The identification of the metabolic processes during the inflammatory processes would provide new therapeutic opportunities. The study of the T cell metabolism has provided ample resources regarding the pathways important for the T cell plasticity and effector functions (42). The metabolic demand of every immune cell depends on the particular function it performs, this is evident in the subsets of CD4+ T cells where effector and Th17 cells rely on aerobic glycolysis while memory T cells and Tregs rely on fatty acid oxidation to produce energy (42). The other activated immune cells, such as dendritic cells, neutrophils, and pro-inflammatory macrophages, rely on aerobic glycolysis for energy. During activation, T cells increase their glucose uptake through Glut1, which facilitates increased oxidative phosphorylation and glycolysis to sustain proliferation of these cells (42). The two main biochemical pathways that lead to generation of adenosine triphosphate (ATP) and the metabolic precursors for biosynthesis of immune cells includes glycolysis and tricarboxylic acid (TCA) cycle (43). In proliferating cells, glucose is broken down to pyruvate by glycolysis, which is further oxidized by the TCA cycle in mitochondria (43). The study by Michalek et al. (44) determined that pro-inflammatory cells displayed a stronger bias toward glycolysis whereas the induced regulatory cells displayed mixed metabolism, including glycolysis and lipid oxidation. Since, nutrients affect metabolic changes, which in turn affect the differentiation state of the immune cells, dietary interventions could be used to cause metabolic changes in a response to infection.

Malnutrition is an important example of immunosuppression caused due to macronutrient and micronutrient deficiencies in our immune system (7). It predisposes individuals to infection by impairing the integrity of epithelial cell barrier and suppressing the immune responses (45). Enteroaggregative *E. coli (EAEC)* infections causes diarrhea-like symptoms in immune-compromised individuals and particularly in severe cases in children with malnutrition. A study by Philipson et al. (46) demonstrated that malnourished mice exhibited an impaired ability to induce pro-inflammatory cytokine during the *EAEC* infection. The observed immunodeficiency of the mice demonstrated that the malnourished mice were unable to mount protective innate or adaptive immune responses against *EAEC* infection (46). Another study by Philipson et al. (47) showed that tryptophan is a crucial element for antibacterial protection against infection. Mice fed with tryptophan-free diet had reduced antimicrobial peptide production against the high *EAEC* pathogen levels. A study by Bolick et al. (48) demonstrated that zinc deficiency impaired the immune responses in response to *EAEC* by increasing the virulence factor associated with it. The zinc-deficient mice challenged with *EAEC* had greater weight loss, mucus production, and diarrhea compared to the control group. The nutritional supplements and interventions, such as vitamins and mineral supplements, polyunsaturated fatty acids (PUFAs) have been studied extensively over the past decade (49). Additional breakthrough studies include the association between vitamin E and T cells, vitamin A, and mucosal immunity, role of zinc in T and B cell development and the effect of PUFAs composition of the diet on inflammation and immunity. A study by Meydani et al. (49) demonstrated the reversing effect of vitamin E on age-associated defect in T cells. Vitamin E enhances the T cells via a direct effect on T cells and an indirect effect by reduced production of PGE2 in macrophages (49). Furthermore, several studies have shown that retinoic acid (RA), a major oxidative metabolite of vitamin A, plays a key role in the differentiation of T cell subsets, migration of T cells into tissues and their regulatory function (50) that provides further evidence for the role of vitamin A in mucosal immunity. Adequate vitamin A status in animal models, whether derived from ingestion of preformed retinol or β-carotene, is important for maintenance of the proper balance of well-regulated T cell functions and prevention of excessive or prolonged inflammatory reactions. In addition, zinc deficiency (51) has been shown to be partially responsible for increased apoptosis of pre-T cells; and also crucial for the balance between the different T cell subsets. Accordingly, zinc supplementation restores the Th1/Th2 balance; however, high dose of Zn^+2^ reduces the development of Th17 cells. Furthermore, zinc deficiency is known to cause the reprograming of immune system that accelerates apoptosis among premature and immature B cells, and causes decreased antibody production due to the chronic production of glucocorticoids (52). Another, important component includes the proportion of different types of PUFAs present in the diet and its effect on immune cell functions. The dietary n-3 PUFAs present in fish oil modulate immune responses and the expression of transcription factors involved in controlling inflammation (53–55). Dietary n-3 PUFAs also aid in the suppression of pro-inflammatory cytokines produced by the macrophages and reduce the symptoms of animal models of autoimmune disease (24).

These studies show the effect of various dietary components on the immune system. However, a global mechanistic understanding of the interplay between infection, microbiome, metabolism, and nutrition is currently lacking.

The direct alteration of mucosal communities by the nutritional interventions has led to the evolution of nutritional immunology, leading to advancement in the field of medicine. One such “use case” for the effective use of nutritional-based intervention is the use of CLA in the treatment of immune-mediated inflammatory disorder of the gastrointestinal tract, such as Crohn’s disease (CD). CLA is a mixture of positional and geometric isomers of octadecadienoic acid. The use of CLA has been explored due to numerous anti-inflammatory and anti-oxidant properties that have been characterized in animal models (56–58). Dietary CLA supplementation has been shown to suppress colonic inflammation in pigs with bacterial-induced colitis (58) by the up-regulation of the colonic PPARs expression. CLA decreased the disease severity of experimental IBD in pigs by activating colonic PPAR γ (58). Another mechanistic theory proposed to explain the benefits of dietary CLA includes inducible eicosanoid suppression in the endoplasmic reticulum. CLA has also been shown to ameliorate inflammation-driven colorectal cancer in mice (59) and has enhanced cellular immunity by modulation of the effector function of CD8+ T cells and antiviral responses in pig models (60, 61). It is a unique compound known to exhibit anti-inflammatory effects along with stimulating cellular and adaptive immune responses to bacterial and viral infections.

The immunomodulatory efficacy of CLA was tested in patients with mild-to-moderate CD in an open-label study for 12 weeks (62). Oral CLA administration was well tolerated in these patients, and CLA suppressed the ability of the peripheral blood T cells to produce pro-inflammatory cytokines, such as interferon gamma (IFN-γ), tumor necrosis factor-α (TNF-α), and IL-17. The study demonstrated decreased CD activity index and increased quality of life of patients with CD (62). It also provided insights on possible mechanisms of immune modulation by CLA, a nutritional intervention targeting the human system (62). The patient level data obtained from the clinical study was used as a training dataset to develop a larger synthetic population for *in silico* experimentation of the Phase III placebo-controlled, randomized clinical trial (63). The study (63) demonstrated that post-treatment highlighted a positive correlation between the initial disease activity score and the drop in Crohn’s disease activity index (CDAI) score. It highlighted the need for precision medicine strategies for IBD treatment, wherein treatments specific to an individual would yield better outcome as opposed to the one size fit all strategy.

Another “use case” for nutritional immunology research is abscisic acid (ABA), a plant phytohormone, which when used as a dietary component elicits immunomodulatory properties. A benefit of dietary ABA-supplementation in mice includes anti-diabetic effects, anti-atherosclerotic, and an anti-hypersensitive effect that has been shown in various studies (64–66). The study by Guri et al. (64) showed that ABA improved insulin sensitivity and reduced adipose tissue inflammation when supplemented into diets of obese mice. Another study by Guri et al. (65) showed that mice treated with 100 mg/kg of racemic ABA mixture significantly reduced recruitment of CD4+ T cells in the aortic root (67). ABA has also been identified as a ligand of lanthionine synthetase C-like 2 (LANCL2), a novel therapeutic target. A study by Hontecillas et al. (68) investigated the immune modulatory mechanisms underlying the anti-inflammatory efficacy of ABA against influenza-associated pulmonary inflammation. When ABA was given preventively or therapeutically, it ameliorated the influenza virus-induced pathology by the activation of PPAR γ in pulmonary immune cells, along with suppression in the initial pro-inflammatory responses and promoted resolution of the infection. A recent study by Magnone et al. (69) showed that the mechanism by which low dose of ABA (found in fruit extracts or exogenous) lowers the blood glucose level does not involve insulin release at all. They showed that ABA had a lowering effect on glycemia without having an effect on insulin concentration in the blood. The study focused on finding the bioavailability of dietary ABA mainly the one found in fruits (apricots primarily used in the study) and the effect of these fruits in general on glucose tolerance. The rats and human fed with fruits extract (with ABA), when compared to the control group had lower glycemia and insulinemia. When a dose ABA was administered orally without fruit, an equivalent dose of ~1 ug/kg (69) successfully lowered glycemia and insulinemia during the oral glucose tolerance test. The mean glycemia with the fruit extract was significantly lower than the exogenous ABA. The lowering effect of ABA on glycemia lasted for at least 6 h after intake (69), showing that it contributed toward disposal of glucose in the blood. The results also showed that apricot extracts increased ABAp (ABA plasma levels) higher than glucose did, which led them to the conclusion that high bioavailability of oral ABA can be obtained from the fruit extracts. The mechanisms by which this plant hormone and secondary by-product of soil fungal metabolism regulate glucose metabolism and immune responses in humans remain largely unknown.

The research on the role of single nutrients in immune functions is extensive; however, this is not the case for multiple nutrients and the existing combinatorial effect of interactions between the various nutrients remains largely unknown. The interactions between multiple nutrients can negatively affect the immune system, for example, excess of calcium interferes with leukocyte function by displacing magnesium ions, causing reduction in cell adhesion processes (70). The nutrient deficiencies can either singly or combinatorialy affect the host immune system in multiple ways. The regulation of the immune system by the nutrients can either be beneficial or detrimental. For example, the nutrients involved in antimicrobial and antitumoral function of macrophages can be modified by nutrients that promote synthesis of reactive oxygen or nitrogen intermediates (70). A recent study by Lacroix et al. (71) showed how systems biology methods can be applied to better understand the potential role of nutritional interventions, such as caloric restriction and polyphenol supplementation to promote health aging processes and reduce metabolic risk factors. Thus, although a comprehensive level understanding of the complex mechanisms underlying the combinatorial effect of nutrients is challenging, a systems-wide approach integrated with computational modeling and informatics can aid in elucidating this complex process.

## The Current Approaches and the Urgent Need for Paradigm Shift

### Understanding Reductionist Approaches toward Nutritional Immunology

Traditional reductionist nutritional immunology approaches have prevailed in the field and focused on studying the interplay between nutritional deficiency or supplementation and their effects on specific parts of the system while disregarding global effects. Until recently, researchers have only been able to extrapolate data that involved a subset of nutrients and their gene interactions, along with the key pathways of the immune system. A comprehensive systems-wide understanding of any biological system requires the harnessing of data that include genes, proteins, RNAs, their interactions, changes in concentration, and regulation under certain conditions (72). Traditional approaches are based on reductionist methods alone, which do not take into account that systems are a part of greater networks of interacting entities, including genes and nutrients. However, with the advent of fields such as Nutrigenomics (73) and Nutrigenetics, the field is slowly advancing toward using the tools initially developed for genetics research. However, when analyzing massively interacting systems, such as the relationship between nutrients, microbiome, metabolism, and immune response, there is a need for computational modeling techniques (74). Nutrigenomics and Nutrigenetics refer to the interface between nutritional environment and their interaction with cellular and genetics approaches. The development of novel sequencing tools in these fields of nutritional science focuses in determining the overall effect of nutrition on the human genome and the modulation of several molecular mechanisms that affect different physiological functions inside a human body. The advancements in genomics have resulted in incremental knowledge discovery that takes into consideration: how an individual’s genome expresses itself at different *omic* levels (proteomics, metabolomics, lipidomics) in response to nutrition. An effort toward post-genomics data, and multi-omic data integration by using modeling provides a deeper insight of the interaction between our genes, microbiome, and diet. Metabolomics is one of such -omics technology that involves the study of small molecules or metabolites present in the biological samples in order to study the effects on metabolic process under varying biological conditions (75). The study of metabolites yields information about the biological processes since metabolites are implicated in number of human diseases (75). The application of metabolomics in nutritional immunology would include detailed study of alterations caused in metabolic pathway following nutritional interventions. This will allow enhanced understanding of the effect of nutrition on metabolic pathways. The study by Bakker et al. (76) is an example where an integrated metabolomics approach was used to study effects of dietary products that showed anti-inflammatory properties, in a population of overweight men. The profiles of gene expression, proteins, and metabolites were integrated with the measures of inflammation markers and the results, obtained after integrated omics approach, demonstrated that the dietary products modulated inflammation and oxidation with alteration in the metabolism status of the healthy overweight men. A more transformative approach that would include information-processing representations of nutritional immunology is required to tackle the challenges in this field. This would involve using interdisciplinary approaches from computer science, systems modeling, bioinformatics, and data science for big data analysis, which would allow researchers to reverse-engineer the system. Thus, the application of systems biology methods in nutritional immunology research has the potential to accelerate the discovery of novel biomarkers and systems-level mechanistic understanding of how nutrition modulates our immune system and health outcomes. One key step in this iterative process is the validation of modeling-derived predictions that require targeted pre-clinical, mechanistic, or clinical studies. This step represents the confluence between systems-level analyses and the need for reductionist validation studies.

### Systems Nutritional Immunology: A Systems-Level Approach to Nutrition–Microbiota–Immune System Interactions

**Figure 3:**
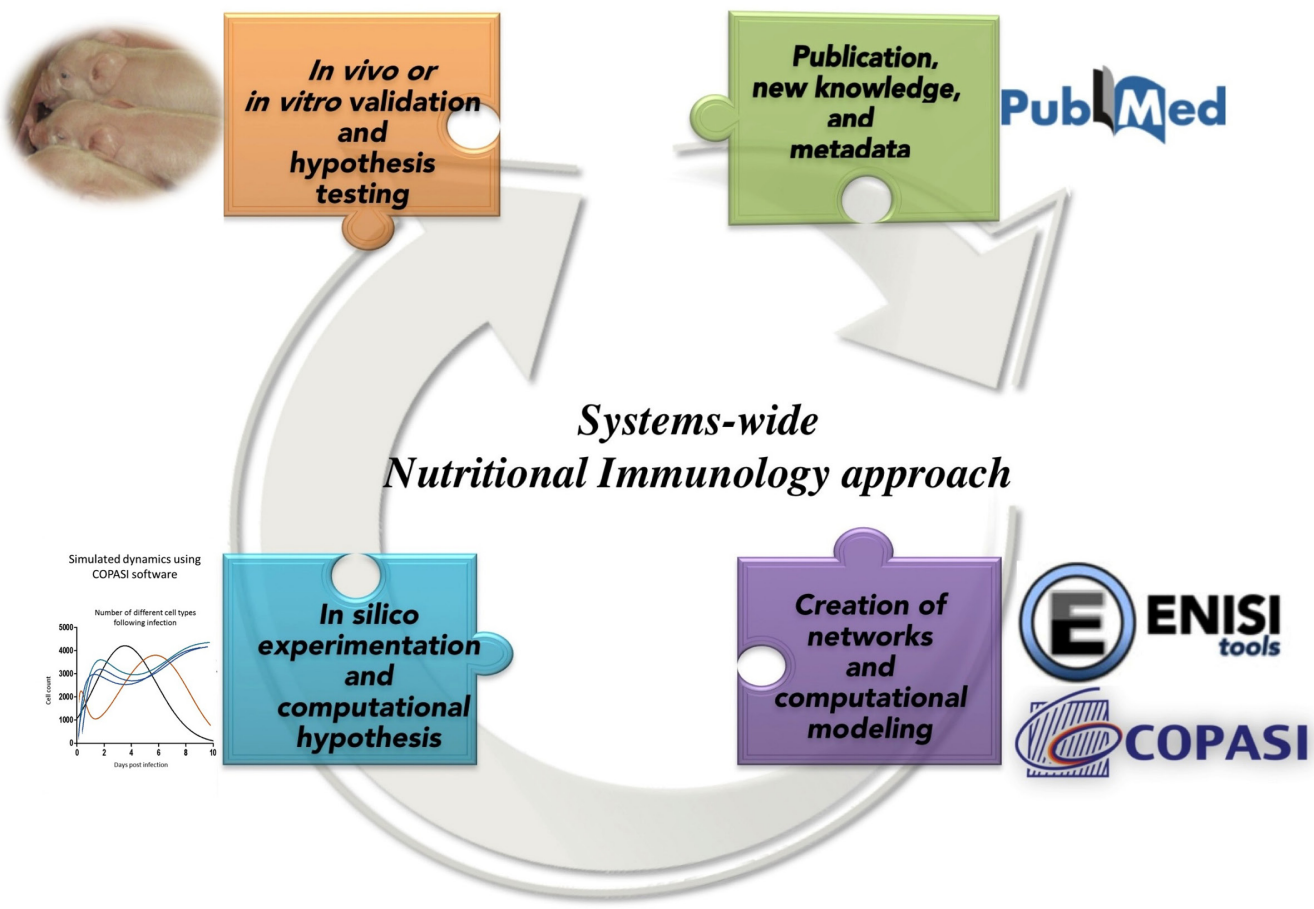
**Integrated information biology methods applied to Nutritional Immunology**.

The Modeling Immunity to Enteric Pathogens project (MIEP)^1^ and the Nutritional Immunology and Molecular Medicine Laboratory^2^ are examples of successful implementation of modeling approaches for the study of complex mucosal immune responses in the context of infectious diseases. Under the MIEP project, a first step toward building information-processing representations of the mucosal immune system was undertaken. However, similar initiatives are lacking in the field of nutritional immunology or for chronic and autoimmune diseases. Computational modeling in combination with big data analytics, portal science, and informatics, enabled by high-performance computing (77–79), is essential components in the study of massively interacting systems, such as host immune response–gut microbiota–nutritional interactions. As proposed in *Goals in Nutrition science 2015-2020* (74), a mechanistic understanding of the host–nutrient–microbiota interactions enabled through computational modeling based on integrated information biology methods have an enormous potential to predict the outcomes of the nutrient-microbiot-immune systems interactions as shown in Figure 3.

The main challenges in systems biology frameworks are the complexity of systems and the output in terms of vast amount of data with scattered pieces of knowledge that needs to be connected together and be made sense of. The need for development of computational tools becomes imperative for the integration of the data (80). The advent of user-friendly tools for informatics, modeling, and advanced big data analytics enables the prediction of emerging global behaviors of biological systems and the characterization of novel molecular and cellular mechanisms (80).

Over the past decade, a significant increase in computational power and availability of larger experimental datasets has allowed models to be more comprehensive and in some cases multi-scaled (78, 81–84). In addition, development of software workflow, such as Epidemic Simulation Systems (EPISIM) (85) has facilitated the semantic model integration for biologically skilled scientists especially with the growing number of available models [over 163 nutrition themed systems biology markup language models (SBML)] that are already available in the Biomodels database (86). The methodologies to extract actionable knowledge from such rich data and metadata have been facilitated by the development of standards [such as SBML (87) and more recently Markup language for Allergens (AllerML) (88), ontologies (89), for example, Medical Subject Headings (MeSH) (90), Unified Medical Language System (UMLS) (91), and Gene Ontology (GO)], and curated specialized databases such as Therapeutic Target Database (TTD) (92), hepatotoxicity database (93), drug combination database (94), Food and Drug administration (FDA) toxicity databases, real-time data entry (95), and White adipose tissue reference network (WATRefNet) (96). New bioinformation technologies combine relevant models and data to address important questions whereby the answers reside in the interface between networks.

#### Computational Tools

Computational tools and big data help revolutionize biological research in a way that is shifting the paradigm from top-down or bottom-up approaches to a middle-out approach. The latter is based on conceptualizing models and tools at the level that provides the richest data and connection that to higher or lower levels for comprehensive integrated systems. Building massively interacting multiscale models (MSM, theoretical and data-driven) anchored around unanswered immunological questions holds a promise for the advancement of the field of nutritional immunology into unprecedented scientific discoveries. A recent work by Ramsundar et al. (97) investigated the aspects of multitask learning with an application to virtual screening. The multitask networks trained on 40 million experimental measurements for more than 200 targets showed significant improvements in comparison to the basic machine-learning methods (97). Their findings show that the amount of data and tasks both had an impact on the outputs. The efficacy of multitask learning was directly correlated to the available relevant data, which emphasized the concept that bigger data are of critical importance for improved predictions (97). Furthermore, metabolomics studies can be integrated, through rigorous methods, with biochemical, metabolism, and immunological networks in order to form more comprehensive pictures regarding the complex systems-level interaction. Immunometabolism data include changes in metabolite composition and immunological parameters that can aid in studying the interaction between nutrients, immune system, and microbiome that changes during the progression of a disease. Such data and theory can be used to build computational models with an inclusion of the interaction effects of nutrients, such that the model can be calibrated with large time-series multi-omic datasets. The outputs obtained from the models can be integrated with the experimental studies along with inclusion of molecular modeling techniques, such as molecular docking approaches. The docking studies can determine how nutrients modulate the various metabolic and immunological networks that can be experimentally validated with surface plasmon resonance (SPR) spectroscopy. For example, a better understanding of the changes in metabolites caused during the varying nutritional demands of the immune cells, which includes – the reliance of effector and Th17 cells on glucose and memory T cells and Tregs on fatty acid oxidation can elucidate – (1) how a set of nutrients modulate biochemical pathways and immune responses of specific cells types and (2) mechanisms underlying the nutritional prevention or amelioration of disease. Systems nutritional immunology can be built with the concept that analyses of multitask networks across nutrition, metabolism, microbiome, and immune system and are required to elucidate emerging mechanistic behaviors that inform health-promoting interventions. In addition to deterministic models, machine-learning methods can also be highly effective in bridging the gap between big data and knowledge. For instance, it has been shown that supervised learning methods, such as artificial neural network (ANN) or random forest (RF) can be alternative solution to ODE-based modeling (63, 98–100), and can more efficiently be used to model complex systems, such as the CD4+ T Cell differentiation (98). Unsupervised method can also be valuable in knowledge discovery as they allow deeper analysis of large datasets and can be instrumental in developing mechanistic models as well candidate gene prioritization, and overall understanding of the complex intertwined systems (101, 102).

#### Systems Biology Tools: Contribution of Agent-Based Modeling and Multiscale Modeling

With the continuous generation of massive amount of data, there is an urgent need to integrate big data, theory, procedural knowledge, and mechanistic information to synthesize and simulate recognizable behaviors of massively interacting systems. Mathematical modeling and simulation are the techniques that can be utilized for dynamic knowledge testing. Models have the power to discover new findings through effective computational technologies. The knowledge acquired from the computer simulations can form a formal basis of testing the finding in the lab, and validate the known findings (103). The two major categories of modeling technologies include equation-based and agent-based modeling, with a limited number of equations, mathematical models provide an efficient solution; however, it is challenging to incorporate the biological processes in the mathematical equation. Agent-based modeling (ABM) on the other hand uses agents to represent the units in the biological processes (103) wherein these agents follow certain rules and have unique properties that represent different states of the biological entities, such as their location (84), genotype, and movement. The enhanced capability of ABMs can simulate extremely complex biological behaviors for which the requirement of high-performance computing (HPC) is a must (104). Traditional ODE methods and ABMs provide useful information. However, analysis of the complex nutrient–immune system–microbiome interactions and study of how these interactions change over time requires an understanding of all the key components at varying scales in space that include molecular to tissue level, population level scales, and time from nano-seconds to year (105). This necessitates computational modeling across spatiotemporal scales. The advancement in computer hardware, algorithms, as well as computational power has contributed to the development of multiscale models (MSM).

The MIEP has developed the Enteric immunity simulator multi-sale modeling (ENISI MSM) (78, 84), the first agent-based simulator for enteric immune systems. ENISI MSM integrates five orders of spatiotemporal scales and is based on both deterministic (ODE and partial differential equations) and agent-based models, integrated in a single unit. It is designed specifically for application in computational immunology along with strong visualization module for the representation of the tissue level scale in the MSM system. ENISI MSM allows the combination of different tools, techniques, and modeling strategies thereby integrating diverse types of data across different scales along with sensitivity analysis in order to validate the model-driven hypothesis with experimental data. With respect to components, the ENISI MSM model can stimulate signaling pathways, metabolic networks, cytokine diffusions, cell movement, and tissue modeling (84). A multiscale CD4+ T cell differentiation model when calibrated with experimental data and tested in the context of gut inflammatory was able to produce *in silico* experimentation that was used to study the complex host–pathogen interactions as well as host–nutrient–microbiota actions (106). ENISI Visual (104) provides a user-friendly interface for users to change the number of immune cells and to observe simulation speed. The MSM tools can be utilized in the field of systems nutritional immunology, wherein the effect of nutritional components on the immune cell parameters can be modeled and modified accordingly. ENISI MSM is a tool designed for modeling the mucosal immune responses that can simulate 10^7^−10^11^ cells in high-performance simulations (78, 84, 104, 107–110). The high-performance computing-driven ENISI MSM enhanced the development of massively interacting models of the mucosal immune system and significantly increased the power of *in silico* experimentation with a scalability of 10^9^−10^12^ (106). While the tool was initially developed to address problems related to infectious and immune-mediated diseases, ENISI can be adapted to develop new information-processing representations of host–microbiota–nutrient massive interactions.

#### *In Silico* Techniques – A Nutritional Immunology Revolution

Traditional tools, such as *in vivo* and *in vitro* models have been consistently used in order to test hypothesis and perform quantitative studies. However, traditional reductionist experiments have led incremental knowledge generation due to the abundance of reductionist approaches. Over the past decade, new computational techniques, such as *in silico* methods (111), have been applied to address the failures in trials for Alzheimer’s disease (112) and the clinical trial related to trauma-induced critical illness (113). The *in silico* methods are based on quantitative relationship between the parameters, and include homology modeling, machine-learning, data mining, network analysis tools, and data analysis tools that require high computational power and capabilities (111). For instance, *in silico* pharmacology is a rapidly growing new field that incorporates the newly developed techniques in order to integrate patient clinical data. It involves the development of computational models based on certain algorithms to make predictions, propose new hypotheses, and advance toward new horizons in medicine and therapeutics. *In silico* clinical trials provide an opportunity to develop synthetic population and conduct large-scale clinical trial simulation thereby aiding in the design and testing of new nutritional components. The application of *in silico* methods has also been utilized in the complex process of drug discovery. The review by Ekins et al. (111) describes various *in silico* methods for pharmacology that are being utilized in the drug discovery process. The process of “virtual screening” involves scoring and ranking the molecules in large chemical libraries according to their strength of affinity to a certain target (111). Thus, the valuable information provided by the power of *in silico methods* can be extended beyond the fields of immunology and pharmacology and be applied to systems nutritional immunology in order to predict the outcome of dietary interventions on the human health.

The concept of *in silico* trials provides insights and guidance into the design of clinical trials of immunomodulatory therapies, especially the ones that have severe side effects. The process ranges from optimal patient selection to individualized dosage and duration of proposed nutritional/therapeutic intervention (114). Machine-learning algorithms or ABMs can be utilized to create synthetic patients from existing clinical trials (63).

##### Case Study

One of the “use cases” that explains the success of *in silico* experimentation is the identification of lanthionine synthetase component cyclase-like 2 protein (LANCL2) and its application as a treatment option for the *in silico* clinical trial. MIEP performed series of modeling studies that included computational-based drug design methods (115), biochemical and *in vivo* studies (116) to confirm LANCL2 as a novel and promising target for the discovery and development of orally active, broad-based drugs against inflammatory, immune–mediated, and chronic metabolic disease. The *in vivo* studies comprised of using LANCL2 ligands as a treatment option on human peripheral blood mononuclear cells that showed a significant drop in the inflammatory and proinflammatory cytokine levels (116). The results were validated in mouse models of IBD in which LANCL2 ligands were used as an alternative treatment for CD patients (116). Based on the preliminary results and experimental data, MIEP used advanced machine-learning algorithms to create a large synthetic population of CD patients and designed a Phase III clinical *in silico* clinical trial study (63). The synthetic patients were randomly allocated to different treatments under the study and the effectiveness of these treatments were analyzed based on the changes in CDAI scores (63). The results from the study provided an insight that the efficacy of LANCL2 therapeutics can be extendable to all stages of CD.

Similar approaches can be designed and extended to gain new insights of the interactions between diet, genetic factors, microbiome populations, and response to treatment to precision medicine intervention. The effect of treating the set of synthetic population with biologics, therapeutics, pharmaceuticals, nutritional components, or combinatorial interventions (i.e., nutritional adjuncts along with therapeutics) can be examined. The output can provide valuable data for accelerating drug development pipelines with improved capability to predict the likely response to any treatment (63, 114).

## Challenges in Computational Capabilities

Despite the fact that *in silico* experimentation and modeling have the capability to yield basic insights and translational applications in critical illnesses, many challenges still remain in this rapidly evolving field (20). The key challenges involved in modeling the biologically complex systems are that they encompass many levels of systems and scales. The inherently multiscale, multi-system, multi-network nature of critical illness adds on to this complexity. The challenge also comprises the integration of nutrition, immunological, metabolic, and physiologic processes required to decipher the multi-compartment and multi-dimensional landscape of biological systems (20). Furthermore, the process of building mathematical models of complex biological processes and their computer simulation is an iterative one. The initial information-processing representation of the *in vivo* counterparts would incorporate genomic, biochemical, microbial, immunological, and physiological data (117) that would eventually require the data calibration from experimental counterparts. The major challenges that would arise include explosion of massive time-series biological data, with increasing demand for storage of the larger datasets and computational power. This would require the development of new tools and techniques for the management of knowledge from the biological datasets. Furthermore, given that analysis and visualization of the massive data is complicated, the importance of open science becomes increasingly important (118). Data availability would enhance the progression of data analysis procedures. It is important to understand the power of big data and the analysis process, as it adds knowledge to the existing hypothesis (118). However, the high dimensionality of big data puts forth computational and statistical challenges that include scalability, storage bottleneck, computational cost, and algorithm instability (119). In order to extract the knowledge and exploit parallelization of computation, skilled programmers and bioinformaticians need to adopt to the new programing platforms, tools, and practices such as in memory processing, graph databases, and advanced machine-learning algorithms (120). Along with modeling and informatics tools, a web portal that facilitates the collection and integration of experimental and computational data and metadata along with analysis processes, model building and quality assurance are needed to support precision medicine interventions. Therefore, it is challenging to not only overcome the hardware bottlenecks and software complexity, but also to keep up with the ever-changing technological advancements and the need for seamless integration.

The multiscale models represent different spatiotemporal scales with distinct spatiotemporal properties. This increases the need to improve the computational performance and synchronization across scales. In a hybrid approach, for example, ENISI MSM (84) calls the sub-models in the different scales in each simulation cycle, and the final output can be the integration of the outputs form each scale. Since, the ODE solver Complex Pathway Simulator (COPASI) (121, 122) used in ENISI MSM is a large object, loading millions of objects with different scales significantly slows the simulations, due to high memory processing activities. The implementation of the system with the use of high computational power for model simulations can aid the analysis of increased number of realistic number of agents required for *in silico* studies. Also, there is a need for improvement in enhancement of the visualization components of the models that can aid the adaptability of the system among experimentalists. The solutions designed to address the challenges related to the tools used in computational immunology can be extended to develop bio-information systems, models, web portals, and tools adaptable to systems nutritional immunology research.

## Developing Information-Processing Representations of Systems Nutritional Immunology

The Modeling Immunity of Enteric Pathogens project^1^ has successfully developed user-friendly tools and models to characterize the mechanisms of immunoregulation underlying immune responses to enteric pathogens. The MIEP technology is HPC-driven as illustrated by ENISI MSMv2, a tool that models mucosal immune response and scales up to 10^11^ agents in HPC simulations (106). This is an important hallmark achieved toward building large-scale information-processing representation of immune response at multiple levels. Overall, the HPC-driven ENISI MSM platform combines the study of molecular pathways controlling T cell differentiation and tissue-level interactions between cells with an aim to characterize novel mechanisms of immunoregulation at the gut mucosa. MIEP is also working toward the integration of bioinformatics, computational modeling, and experimental validation in order to study the mechanisms of tolerance during bacterial and viral infection. These modeling-driven predictions have the potential to accelerate the scientific discovery process.

Notable MIEP-based achievements include (1) development and enhancement of a suite of tools for ABM (ENISI)- and ODE (COPASI)-based modeling of immunological processes (79, 104, 110, 123); COPASI supports models in the SBML standard and can import and export models in the SBML format. The software allows to perform simulations either with stochastic kinetics or with differential equations and can be used to perform, analysis, sensitivity analysis, and user-friendly data visualizations; (2) development of validated computational models of CD4+ T cell differentiation and function (77, 124, 125), mucosal immune responses to *Helicobacter pylori* (126), modulation of CD4+ T cell responses to *H. pylori* by IL-21 (125); (3) development of mouse and pig models of *H. pylori* infection (127, 128) and mouse models of EAEC infection (46, 129, 130); (4) development of a methodology allowing the creation of dynamic models combining theoretical knowledge and time-course high-dimensional datasets; (5) initial characterization of the ability of *H. pylori* to induce CD8+ T cell responses in the pig model; (6) determination of the role of *H. pylori*-infected mononuclear phagocytes on the modulation of mucosal immune responses to the bacterium; and (7) successful delivery of a summer school and symposium in Computational Immunology (131).

The web portal resources that can be used include the “Immune Modeling Community Web Portal”^3^ where news and relevant resources and news related to immune response modeling for infectious diseases are shared in order to facilitate collaboration and exchange of information between the investigators. Another workflow-based modeling software designed for the use in immunology research is the Differential Equation Modeling Solution (DEDiscover) developed at the Center for Biodefense Immune Modeling. The software can be utilized to perform simulations, parameter estimation, sensitivity analysis, residual analysis, and statistical analysis for a case study represented as a set of differential equations. However, DEDiscover does not fully support SBML and it cannot handle user-defined kinetics on import. Furthermore, it does not export SBML. These shortcomings make COPASI the preferred tool. The Program for Research on Immune Modeling and Experimentation (PRiME)^4^ is another multidisciplinary Immune Modeling Center that is focused on developing (1) mathematical and data-based models for elucidating the viral mechanisms of category A-C viral pathogens and (2) bioinformatics components for data management and model development. The ImmuNet (132), developed by PRiME is a web interface that is aimed to provide immunology researchers with an easy to use resource that can be used to explore the immune-related functional relationship networks. These functional relationship networks provide new mechanistic insights about the previously unknown gene–gene relations and can be used to predict the immune processes associated with any other pathway-relevant components.

To date, most of the nutritional immunology studies have focused on characterizing the effect of nutritional components on individual parts of the immune system, whereas limited effort has been placed on elucidating and modeling the complex, interconnected massively interacting systems. Computational models can be trained using the known effects of the nutrients (data and theory) and the effect of interaction of different nutrients on the massively interacting system can be predicted using advanced machine-learning methods. These predictions can be tested in lab and the deviations can be used to update the models. A key aspect of the nutritional systems immunology cycle is the validation step. That step is inherently reductionist. However, if performed in the broader context of systems nutritional immunology, then the validation studies are guided by the existing theory and data. Another way would include the use of multiscale models wherein the knowledge obtained from multi-omics studies, regarding the regulatory mechanism of nutrition, can be studied with high levels of details ranging from the cellular level to whole body, population, and policy level. The data obtained from the nutritional intervention studies integrated with -omics and targeted modeling-driven mechanistic studies will provide a comprehensive framework to simulate the physiological mechanisms and immunological changes in the body after the intake of nutrients.

A major goal in the advancement of systems nutritional immunology research and development is to build comprehensive, multiscale network models that will accurately predict global and local effects of nutrition-based interventions. The identification of the efficacy of these nutritional-based interventions on the immune system using *in silico* experiments would lead to advancements in research for better treatment for disease mechanisms, assessment of disease risk, and prediction of optimal interventions for the immune-mediated diseases, with an ultimate future goal of expanding the outputs for application in precision medicine. In this review, we specifically highlight the pressing need for the development of predictive systems modeling that provide a comprehensive mechanistic understanding of the system. The conceptual modeling approaches and the computational techniques need to be integrated with advanced big data analytics methods, such as statistical and machine-learning algorithms. In summary, the use of an iterative systems biology cycle of experimental simulation, data collection, along with mathematical and computational model building, simulations, prediction, calibration, refinement, and validation have the potential to gain a systems-level mechanistic understanding in order to guide nutrition-based precision medicine, health, and wellness.

## Author Contributions

Contributed to the design of the paper: JB-R, RH, and VA. Contributed to the writing and reviewing of the manuscript: MV, JB-R, VA, RH, AC, CP, AL, and NT-J. Contributed to the making of the figures: MV, AL, and NT-J.

## Conflict of Interest Statement

The authors declare that the research was conducted in the absence of any commercial or financial relationships that could be construed as a potential conflict of interest.

## Footnotes

^1^www.modelingimmunity.org

^2^www.nimml.org

^3^http://www.imcportal.org/

^4^http://tsb.mssm.edu/primeportal/
